# *Brucella canis* in dogs: An underrecognized zoonotic pathogen requiring a one health response

**DOI:** 10.1016/j.onehlt.2026.101506

**Published:** 2026-06-29

**Authors:** Chiara Storoni, Renato Colognato, Anna-Rita Attili, Silvia Preziuso, Yubao Li, Vincenzo Cuteri

**Affiliations:** a School of Biosciences and Veterinary Medicine, University of Camerino, Via Circonvallazione, 93/95, Matelica, MC 62024, Italy; b Scientific/Biological Consultant, Via XXV Aprile, 251, Ispra, VA 21027, Italy; c School of Pharmaceutical Sciences and Food Engineering, Liaocheng University, N. 1 Hunan Road, Liaocheng, Shandong 252000, China

**Keywords:** *Brucella canis*, Zoonoses, One Health, Dogs, Human

## Abstract

*Brucella canis* is a facultative intracellular, Gram-negative coccobacillus and an underrecognized zoonotic pathogen maintained primarily in domestic dogs. Unlike smooth *Brucella* species, *B. canis* expresses a naturally rough lipopolysaccharide (LPS) phenotype, characterized by absence or marked truncation of the O-polysaccharide chain. This antigenic profile reduces the performances of routine smooth-antigen serological assay and contribute to underdiagnosis in veterinary and human medicine. A recent global meta-analysis including 175,675 canine samples estimated pooled canine prevalence of 7.96%, with substantial geographic heterogeneity and markedly higher values in high-density breeding, kennel, and shelter settings.

In dogs, infection results in reproductive failure, persistent bacteremia, and a high proportion of asymptomatic carriers capable of intermittent shedding. Human infection remains infrequently reported but is likely underestimated; a recent scoping review (68 cases) found that 80% of infections with known exposure were associated with direct dog contact. Clinical presentation in humans is nonspecific, and diagnostic limitations further obscure detection.

This review critically evaluates current knowledge on microbiology, epidemiology, clinical manifestations, and diagnostic challenges of *B. canis*, with emphasis on limitations of existing diagnostic tools, variability in epidemiological data, and gaps in validation of emerging methods. We highlight the need for standardized diagnostic algorithms, improved antigen specificity, and integrated surveillance systems. A coordinated One Health framework is proposed to improve detection, reduce transmission, and strengthen cross-sectoral response.

## Highlights

• Global pooled canine prevalence of *B. canis* is approximately 7.96%, with country-level estimates ranging from <2.5% to >30%.
• Rough LPS lacking O-polysaccharide reduces sensitivity of standard serological assays, contributing to underdiagnosis.
• Human infection is likely underestimated; 80% of documented cases with known exposure are linked to direct dog contact.
• Asymptomatic canine carriers with intermittent bacteremia sustain transmission in kennels and households.

## Graphical abstract

**Figure:**
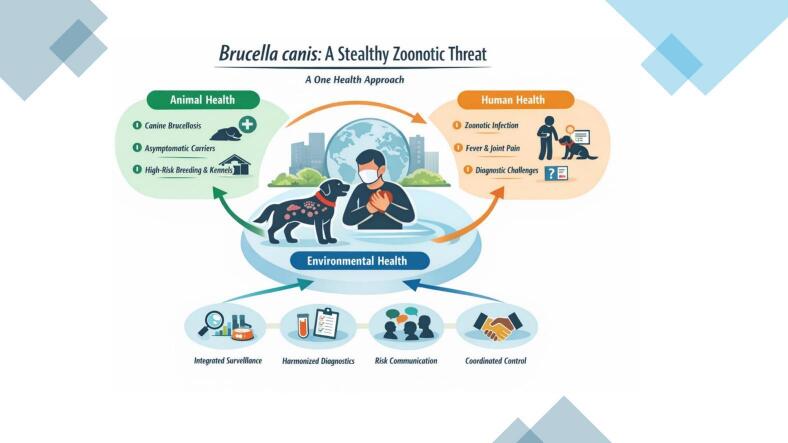

## 1 Introduction

The genus *Brucella* includes several zoonotic bacterial species of major veterinary and public health importance, particularly *B. abortus*, *B. melitensis*, and *B. suis*, which are well-recognized causes of brucellosis in livestock and humans [1]. *Brucella canis*, first associated with abortion outbreaks in beagles in the 1960s, has historically received less attention because it is primarily adapted to dogs and human cases have been reported less frequently [2], [3]. However, accumulating epidemiological, clinical, and diagnostic evidence indicates that *B. canis* should not be regarded solely as a reproductive pathogen of dogs. It is a zoonotic agent maintained at the interface between companion animals, breeding facilities, shelters, veterinary services, households, laboratories, and public health systems [[4], [5], [6], [7]]. (See Table 1.)

**Table 1:** Diagnostic Challenges for *Brucella canis* in Canine and Human Medicine.

Diagnostic method	Principle	Main limitation for *B. canis*	Practical interpretation	Key references
Standard smooth-antigen serology	Detects antibodies to smooth LPS O-polysaccharide	Poor detection of rough *B. canis* lacking O-polysaccharide	Not reliable to exclude *B. canis* infection	[[4], [7], [8], [9], [10]]
RSAT / 2ME-RSAT	Agglutination using rough *B. canis* antigen	Possible nonspecific reactions; antigen quality affects performance	Useful screening tools; positives require confirmation	[[9], [11], [12], [13]]
AGID	Detects antibodies to selected *B. canis* antigens	Variable sensitivity depending on disease stage and antigen	Useful confirmatory component in diagnostic panels	[[9], [11], [13], [14]]
IFAT	Detects antibodies by immunofluorescence	Requires laboratory expertise; validation populations may be limited	Promising confirmatory test; larger validation needed	[15]
ELISA / multiplex assays	Quantitative antibody detection	Commercial availability and standardization remain limited	Useful where validated; not interchangeable across platforms	[[14], [16], [17]]
Culture	Isolation of viable organism	Intermittent bacteremia, slow growth, biosafety risk	Definitive if positive; negative result does not exclude infection	[[7], [9], [18]]
PCR	Detection of *B. canis* DNA	Does not prove viability or active infection; sample dependent	Valuable confirmatory tool when interpreted with other evidence	[[14], [19], [20], [21]]
Clinical presentation	Diagnosis based on signs and exposure	Signs are nonspecific or absent	Should guide test selection, not replace laboratory confirmation	[[7], [9], [11], [22], [23], [24]]

The domestic dog (*Canis lupus familiaris*) is the principal reservoir of *B. canis*. Infected dogs may develop reproductive disease, including infertility, abortion, stillbirth, epididymitis, orchitis, and prostatitis, but many remain asymptomatic while maintaining persistent or intermittent bacteremia [[7], [11], [25], [26]]. This feature is epidemiologically important because clinically normal infected dogs can disseminate the organism within kennels, shelters, breeding colonies, and households. Human exposure occurs mainly through contact with infected dogs or contaminated reproductive materials, although laboratory exposure and contact with urine, blood, saliva, semen, vaginal discharge, aborted fetuses, or placental tissues have also been described [[18], [24], [27], [28]].

The diagnostic challenge posed by *B. canis* is partly explained by its rough LPS phenotype. Smooth *Brucella* species possess an O-polysaccharide side chain that forms the principal antigenic target of many conventional brucellosis serological tests. In contrast, *B. canis* naturally lacks a complete O-polysaccharide chain, resulting in weak or absent detection by tests designed for smooth *Brucella* antigens [[6], [8], [29], [30]]. This antigenic difference contributes to under-recognition in both veterinary and human medicine and complicates surveillance, particularly where species-specific testing is unavailable [[4], [7], [9]].

Recent studies have strengthened the epidemiological case for renewed attention to *B. canis*. A comprehensive meta-analysis reported a pooled canine prevalence of 7.96% across 175,675 samples collected between 1970 and 2025, with marked heterogeneity by continent, country, diagnostic method, and dog population [31]. Higher estimates are frequently reported in kennels, breeding facilities, shelters, and populations with uncontrolled reproduction or high turnover [[31], [32], [33]]. Transboundary movement of infected dogs has also contributed to recognition of *B. canis* in regions where it was previously considered uncommon [[34], [35], [36], [37]].

From a One Health perspective, *B. canis* is important because the infection cannot be controlled by isolated action in a single sector. Veterinary diagnosis affects human risk assessment; human clinical suspicion depends on recognition of canine exposure; dog movement regulations influence geographic spread; and public health response is limited where reporting is not mandatory or standardized [[4], [6], [18], [38], [39]]. Therefore, this review critically evaluates current evidence on *B. canis* infection in dogs and humans, with particular attention to epidemiology, diagnostic uncertainty, treatment limitations, population-level control, and cross-sectoral prevention.

## 2 Microbiology and epidemiology

### 2.1 Microbial characteristics, rough LPS phenotype, and diagnostic implication

*Brucella canis* is a small, non-motile, Gram-negative, facultative intracellular coccobacillus belonging to the genus *Brucella* [[7], [9]]. Like other members of the genus, it can invade and survive within professional and non-professional phagocytes, especially macrophages. Intracellular survival depends on mechanisms that allow the organism to avoid rapid lysosomal destruction, establish a replicative niche, and persist within the host [[40], [41], [42], [43]]. This intracellular lifestyle contributes to chronic infection, intermittent bacteremia, relapse after antimicrobial treatment, and difficulty in achieving microbiological cure [[7], [9], [42], [43]].

A defining feature of *B. canis* is its naturally rough LPS phenotype. In smooth *Brucella* species, the LPS molecule contains lipid A, a core oligosaccharide, and an O-polysaccharide side chain. In *B. canis*, the O-polysaccharide is absent or markedly truncated, resulting in rough colony morphology and altered antigenicity [[6], [29], [30]]. The rough phenotype is associated with defects in genes and loci involved in O-antigen synthesis, assembly, and export, including components of the *wbk* region and related pathways described for rough *Brucella* phenotypes [[6], [30], [44], [45]]. Recent genomic and molecular studies have further clarified the relationship between LPS biosynthesis genes, rough phenotype stability, and host-pathogen interactions in *B. canis* [45].

This LPS structure has important diagnostic consequences. Routine serological assays for human and animal brucellosis often use smooth antigens, particularly those derived from *B. abortus* or other smooth species. Because *B. canis* lacks the O-polysaccharide target recognized by many of these assays, infected dogs or humans may test negative or weakly positive despite true infection [[4], [7], [8], [9]]. Conversely, assays using rough whole-cell antigens can be affected by antigen quality, nonspecific agglutination, and cross-reactions with other rough Gram-negative organisms [[9], [11], [12], [13]]. These limitations require careful interpretation of serology and support the use of combined diagnostic algorithms.

The rough LPS phenotype may also influence host immune recognition. Compared with smooth *Brucella* LPS, rough LPS has different endotoxic and immunostimulatory properties, which may contribute to atypical inflammatory responses and chronic persistence [[6], [30]]. However, the biological meaning of “stealth” should not be overstated. In this review, the term refers specifically to four measurable features: asymptomatic carriage, intermittent shedding, intracellular persistence, and diagnostic under-recognition. It does not imply absence of virulence or absence of clinically important disease.

### 2.2 Epidemiology, regional distribution, and risk factors

*Brucella canis* has a global distribution, but reported prevalence differs substantially among studies. This heterogeneity reflects true differences in dog populations and management systems, but also differences in diagnostic assays, sampling strategy, case definitions, and surveillance intensity [[31], [46]]. Therefore, prevalence estimates should be interpreted cautiously and in relation to the tested population.

A recent meta-analysis including 175,675 canine samples estimated a pooled global prevalence of 7.96% [31]. At continental level, higher pooled estimates were reported in North America, Africa, and South America, whereas lower overall estimates were reported in Europe [31]. However, low estimates in a region should not be interpreted as evidence of absence, especially where surveillance is limited or where testing is performed mainly after clinical suspicion. At country level, estimates range from below 2.5% in some settings to above 30% in others, reflecting differences in endemicity, population structure, diagnostic method, and control practices [31].

High-risk canine populations include commercial breeding kennels, shelters, rescue populations, imported dogs, and dogs from environments with uncontrolled breeding [[31], [32], [33], [34], [36]]. Outbreaks in breeding kennels are particularly difficult to control because transmission may occur before clinical signs are recognized, and asymptomatic infected animals may remain within the population [[25], [33]]. In shelters and rescue systems, high turnover, unknown reproductive history, incomplete testing before movement, and close housing increase the opportunity for exposure [[9], [32], [34]].

Transmission among dogs occurs through multiple routes. Venereal transmission is highly efficient because infected males can shed bacteria in semen, and infected females can shed organisms in vaginal secretions, especially during estrus, parturition, or abortion [[7], [11], [25]]. Late-term abortion, stillbirth, placentitis, and vaginal discharge are major sources of environmental contamination [[47], [48], [49], [50]]. Oronasal exposure occurs when dogs ingest or lick contaminated reproductive tissues, fluids, bedding, food bowls, surfaces, or fomites [[7], [27]]. Vertical transmission can occur *in utero* or through infected milk, and surviving puppies may appear clinically normal while remaining infected [[27], [47]].

Human-mediated movement of dogs is a major driver of geographic dissemination. Importation, rescue transport, breeding exchange, and interstate or international trade have been linked to spread of *B. canis* into new regions [[34], [35], [36], [37], [51], [52]]. In the Netherlands, imported infected dogs were associated with transboundary spread between 2016 and 2018 [34]. In Italy, the first isolation from a breeding kennel highlighted the potential for recognition of infection in areas where surveillance had been limited [35]. Similar concerns have been reported in North America and northern Europe [[33], [36], [37]].

Wild canids and non-domestic hosts may occasionally be infected, but domestic dogs remain the primary reservoir relevant to household and occupational exposure [[27], [53]]. The relative contribution of wildlife to maintenance of *B. canis* is less well defined than the role of domestic dog populations and requires further study.

## 3 Clinical disease in dogs

### 3.1 Reproductive disease

Canine brucellosis caused by *B. canis* is classically associated with reproductive failure. In females, infection may cause late-term abortion, stillbirth, embryonic death, weak puppies, infertility, prolonged vaginal discharge, and placentitis [[7], [11], [19], [47], [48], [54], [55], [56]]. Aborted fetuses, placentas, and reproductive fluids may contain high bacterial loads and represent important sources of infection for other dogs and humans [[47], [48], [49], [50]].

In males, clinical manifestations include epididymitis, orchitis, prostatitis, testicular atrophy, scrotal dermatitis, infertility, and abnormal semen quality [[7], [11], [37], [57], [58]]. Shedding in semen is epidemiologically important, especially in breeding animals. Urinary shedding may also occur, particularly in intact males with involvement of the prostate or reproductive tract [[7], [11], [25]].

### 3.2 Extragenital and systemic disease

Although reproductive disease is central to transmission, *B. canis* should also be considered in dogs with systemic or extragenital manifestations. Reported signs include lymphadenopathy, lethargy, weight loss, fever, lameness, uveitis, and discospondylitis [[18], [22], [23], [50], [59], [60]].

Discospondylitis is one of the most important extragenital manifestations of canine brucellosis. Dogs with discospondylitis may show spinal pain, reluctance to move, lameness, neurologic deficits, vertebral lysis, and narrowing of intervertebral disc spaces [[22], [23]]. Because discospondylitis can occur in the absence of overt reproductive signs, *B. canis* should be included as a key differential diagnosis in compatible cases, particularly when there is a history of breeding, infertility, abortion, kennel exposure, importation, or contact with dogs of unknown status [[22], [23]]. The revised wording avoids implying that discospondylitis merely “sometimes complicates diagnosis”; rather, its presence should actively prompt diagnostic investigation for *B. canis*.

### 3.3 Asymptomatic carriage and intermittent shedding

A major clinical and epidemiological challenge is asymptomatic infection. Many infected dogs show no overt signs yet remain bacteremic or intermittently shed bacteria [[7], [9], [11], [25]]. Such animals can maintain infection cycles within kennels, shelters, and households. Persistent bacteremia may continue for months or longer, and shedding may occur in semen, vaginal secretions, urine, saliva, milk, aborted materials, and placental tissues [[7], [11], [25], [27], [47]].

Published examples illustrate this problem. Tissue distribution studies in naturally infected canine fetuses and neonates have demonstrated systemic infection in reproductive tissues and offspring [47]. Outbreak investigations in commercial breeding kennels have shown that infected dogs may remain in populations before detection, allowing continued transmission [33]. Serosurveys of shelter and humane society dogs demonstrate that infection can be identified in dogs entering rehoming systems, often without prior clinical suspicion [32]. These findings support routine screening in high-risk settings rather than reliance on clinical signs alone.

## 4 Zoonotic transmission and human disease

### 4.1 Zoonotic relevance

*Brucella canis* is a confirmed zoonotic pathogen. Human infections have been documented in pet owners, breeders, veterinarians, laboratory personnel, children, and immunocompromised individuals [[8], [18], [24], [28], [61], [62], [63], [64], [65], [66]]. Reported human disease is less common than brucellosis caused by *B. melitensis*, *B. abortus*, or *B. suis*, but the apparent rarity of *B. canis* infection should be interpreted cautiously because of diagnostic limitations and low clinical suspicion [[4], [7], [8], [24]].

A recent scoping review identified 68 published human cases of *B. canis* infection [24]. Among cases with known exposure, most were associated with direct dog contact, and reproductive exposure was a prominent risk scenario [24]. This evidence supports the view that human disease is uncommon but likely underrecognized, rather than absent or negligible.

### 4.2 Routes of transmission to humans

Human infection usually occurs through mucocutaneous exposure to infected biological materials. High-risk exposures include assisting with whelping, handling aborted fetuses or placentas, cleaning contaminated bedding, contact with vaginal discharge or semen, and close contact with infected dogs [[18], [24], [27]]. Occupational exposure may occur in veterinarians, veterinary technicians, kennel workers, breeders, shelter personnel, and laboratory staff [[18], [28]].

Needlestick injuries, cuts from contaminated instruments, and laboratory handling of cultures or clinical specimens are additional transmission routes [[18], [28], [39]]. Aerosol exposure is possible during laboratory manipulation or cleaning of heavily contaminated environments, although it is less commonly documented than direct contact [[18], [28]]. Indirect transmission through contaminated fomites is biologically plausible, particularly in kennels and reproductive settings, but the relative importance of this route is less well quantified [27].

### 4.3 Clinical presentation in humans

Human *B. canis* infection generally presents as a nonspecific febrile illness. Reported manifestations include fever, fatigue, malaise, chills, headache, night sweats, lymphadenopathy, splenomegaly, arthralgia, and weight loss [[8], [24], [64], [67], [68]]. In the scoping review, fever was reported in 68% of cases, fatigue or lethargy in 39%, headache in 29%, chills in 26%, and malaise in 26% [24].

Severe or focal complications have been reported, including endocarditis, osteoarticular disease, neurobrucellosis, meningitis, hepatic involvement, epididymo-orchitis, and infection in immunocompromised hosts [[8], [61], [65], [66], [68], [69]]. Although many cases respond to antimicrobial therapy, delayed diagnosis can prolong illness and increase the risk of complications.

### 4.4 Immunocompromised hosts

Immunocompromised individuals may be at increased risk of severe, disseminated, or prolonged infection because effective control of *Brucella* depends on cell-mediated immunity [[65], [66], [70]]. Reported cases in persons with HIV infection demonstrate that *B. canis* should be considered when compatible symptoms occur in patients with dog exposure [[65], [66]]. Risk counseling for immunocompromised pet owners should include discussion of canine testing, avoidance of reproductive materials, hygiene measures, and coordination between veterinarians and physicians.

## 5 Diagnosis: current tools, limitation, and critical interpretation

### 5.1 General diagnostic principles

Diagnosis of *B. canis* infection requires integration of epidemiological risk, clinical signs, serology, culture, PCR, and follow-up testing. No single test is adequate in all clinical scenarios [[7], [9], [11], [12], [13], [14]]. This is particularly important because infected animals may be asymptomatic, bacteremia may be intermittent, antibody kinetics vary over time, and antimicrobial exposure can reduce culture yield.

The diagnostic goal should be clearly defined before testing. Screening high-risk kennel populations, confirming disease in an individual symptomatic dog, investigating human exposure, and documenting infection status before breeding or importation require different approaches and different tolerance for false-positive and false-negative results.

### 5.2 Serological testing

Serological assays remain widely used because they are practical for screening. However, their interpretation is complex. Rapid slide agglutination tests (RSAT), 2-mercaptoethanol RSAT (2ME-RSAT), tube agglutination, agar gel immunodiffusion (AGID), complement fixation, ELISA, immunofluorescence antibody testing (IFAT), and newer multiplex platforms differ in antigen preparation, analytical performance, availability, and validation status [[9], [11], [12], [13], [14], [15], [16], [17], [71], [72]].

Agglutination tests are useful screening tools, especially in high-risk populations, but positive results should not be interpreted in isolation. False positives may occur because of nonspecific agglutination or cross-reactivity with other rough Gram-negative organisms; however, the practical importance of this limitation depends on the test antigen, clinical context, prevalence, and confirmatory method used [[9], [11], [12], [73]]. Therefore, it is more accurate to state that cross-reactivity can occur and that confirmatory testing is required, rather than to imply that agglutination positives are routinely unreliable.

Antigen choice is critical. Assays using *B. canis* M− antigen are preferred for *B. canis* serodiagnosis. Use of *B. ovis* antigen can increase the risk of false-positive or poorly interpretable results because it is not identical to *B. canis* antigenic structure. Carmichael and colleagues emphasized the importance of *B. canis* M− antigen for improved serodiagnosis [81]. Accordingly, this review no longer presents *B. ovis* antigen as equivalent to *B. canis* antigen. Where studies used *B. ovis* antigen, the limitation is explicitly noted.

Recent comparative studies provide useful data but must be interpreted cautiously. Perletta et al. evaluated three serological tests and reported high sensitivity and specificity for selected assays, including strong performance of IFAT [15]. However, the sample size was limited and the population was selected; therefore, these results should be considered promising rather than definitive. Larger, geographically diverse validation studies in naturally infected dogs, asymptomatic dogs, kennel outbreaks, shelter populations, imported dogs, and low-prevalence settings are needed before universal diagnostic algorithms can be recommended.

### 5.3 Culture

Bacterial culture remains the definitive method because it demonstrates viable organisms and enables species confirmation [[7], [9], [18], [74]]. Samples may include blood, semen, vaginal swabs, urine, lymph node aspirates, aborted fetuses, placenta, or affected tissues. However, culture has important limitations: bacteremia and shedding may be intermittent; organism load may be low; prior antimicrobial treatment reduces yield; incubation may be prolonged; and laboratory manipulation creates biosafety risk [[7], [9], [18]]. Culture should be performed in laboratories with appropriate biosafety procedures and awareness of *Brucella* exposure risk.

### 5.4 Molecular detection

PCR can provide rapid and specific detection of *B. canis* DNA from blood, semen, reproductive tissues, urine, or other clinical samples [[14], [19], [20], [21]]. Molecular assays are valuable confirmatory tools, particularly when culture is negative or impractical. However, PCR detects nucleic acid and does not, by itself, prove bacterial viability or active infection. A positive PCR result should therefore be interpreted with clinical findings, exposure history, serology, culture when available, and sampling site. A negative PCR result does not exclude infection because bacteremia and shedding may be intermittent and bacterial DNA may be absent from the tested sample.

Emerging molecular approaches, including isothermal amplification methods such as recombinase-aided amplification, may improve field detection and turnaround time [21]. Nevertheless, analytical sensitivity does not automatically translate into clinical diagnostic accuracy. These assays require validation in larger clinical populations and across sample types before widespread routine use.

### 5.5 Human diagnostic considerations

In humans, diagnosis is particularly difficult because routine brucellosis serology is often optimized for smooth *Brucella* species and may not detect *B. canis* [[4], [8], [10]]. Clinicians should consider *B. canis* in patients with unexplained fever, compatible systemic symptoms, and dog exposure, especially exposure to reproductive materials, infected kennels, imported dogs, or laboratory specimens [[18], [24], [28], [39]]. Blood culture with prolonged incubation, species-level identification, molecular testing, and reference laboratory consultation may be required [[8], [10], [18], [39], [62]].

## 6 Treatment and management

### 6.1 Treatment in humans

Human *B. canis* infection is treated using combination antimicrobial regimens similar to those used for other forms of brucellosis, adjusted according to clinical severity, age, pregnancy status, comorbidities, antimicrobial susceptibility, and specialist guidance [[10], [68], [75]]. Common regimens include doxycycline, a tetracycline-class antimicrobial, combined with rifampicin or an aminoglycoside, typically for at least six weeks, although complicated disease may require longer therapy [[10], [68], [75], [76]].

### 6.2 Treatment in dogs

Treatment of canine *B. canis* infection is challenging. Combination antimicrobial therapy may reduce bacteremia and shedding, but complete eradication is unreliable and relapse is common [[7], [9], [77]]. Reported protocols often include doxycycline combined with an aminoglycoside, fluoroquinolone, or other antimicrobial, but evidence remains limited and heterogeneous [[7], [9], [77]]. Treatment should not be presented as curative unless microbiological clearance is documented over time.

### 6.3 Neutering and shedding

Neutering is an important management intervention because it reduces reproductive transmission and, in males, substantially decreases shedding associated with the reproductive tract and urine. However, it does not guarantee elimination of infection, and infected dogs may still require long-term monitoring, movement restrictions, and owner counseling [[7], [9], [11]].

### 6.4 Management of Kennels and Breeding Facilities

In kennels and breeding facilities, control requires rapid interruption of transmission. Recommended actions include quarantine, suspension of breeding and movement, testing of all dogs, repeat testing at defined intervals, removal or permanent isolation of confirmed positive animals, environmental decontamination, and transparent communication with owners and public health authorities [[7], [9], [11], [25], [33]]. In outbreak settings, “test-and-remove” strategies remain more effective than treatment-based approaches because antimicrobial therapy rarely ensures elimination of infection in dogs [[9], [25], [33]].

### 6.5 Management of Infected Companion Dogs

Management of an infected pet dog involves ethical, emotional, and public health considerations. Euthanasia may be recommended in some guidelines, particularly in breeding kennels, multi-dog environments, or households with immunocompromised individuals, but decisions must consider local regulations, owner capacity, animal welfare, and risk mitigation [[4], [7], [9]]. Where euthanasia is declined or not required, management should include neutering, strict hygiene, avoidance of breeding, prevention of contact with reproductive materials, restriction of contact with vulnerable individuals, repeated diagnostic monitoring, and clear written informed consent.

## 7 One health framework for control

### 7.1 Integrated surveillance and reporting

Surveillance remains fragmented because *B. canis* is not consistently notifiable across jurisdictions [[4], [6], [9], [38], [39]]. Integrated surveillance should include confirmed canine cases, human cases, laboratory exposures, imported dog investigations, kennel outbreaks, and shelter-associated events [78]. Veterinary and public health authorities should share data in a timely manner while respecting privacy and legal requirements.

### 7.2 Harmonized diagnostic algorithms

Harmonized diagnostic guidance is needed for dogs and humans. For dogs, algorithms should combine exposure assessment, reproductive history, serological screening using validated *B. canis* antigens, confirmatory serology, PCR where appropriate, and culture in selected cases. For humans, clinicians should be informed that standard smooth-antigen brucellosis tests may miss *B. canis*, and reference laboratory testing may be necessary when exposure history is compatible [[8], [10], [18], [39]].

### 7.3 Regulation of dog movement

Pre-movement testing should be considered for dogs from high-risk regions, breeding kennels, rescue organizations, and populations with unknown reproductive history [[9], [34], [35], [36], [37]]. International and interstate movement policies should specify acceptable tests, timing, documentation, quarantine requirements, and actions after positive results. Testing protocols must acknowledge diagnostic uncertainty and should not rely on a single negative result in recently exposed dogs.

### 7.4 Professional and public education

Veterinarians, physicians, laboratory personnel, breeders, shelter workers, and dog owners require targeted education. Veterinarians should recognize both reproductive and extragenital manifestations, especially discospondylitis. Physicians should ask about dog exposure in patients with unexplained febrile illness. Laboratory personnel should be alerted when *Brucella* infection is suspected to reduce occupational exposure risk [[18], [28], [39]].

### 7.5 Research priorities

Priority research needs include:

1. Large, standardized prevalence studies in dogs and humans.
2. Validation of serological assays using geographically diverse and clinically representative samples.
3. Development of specific antigens that reduce cross-reactivity.
4. Better evidence on PCR performance by sample type and infection stage.
5. Prospective studies of treatment outcomes and relapse in dogs.
6. Quantification of shedding after neutering and antimicrobial therapy.
7. Risk models for household and occupational exposure.
8. Vaccine development and evaluation.

## 8 Conclusion

*Brucella canis* is an underrecognized zoonotic pathogen maintained primarily in dogs and sustained by asymptomatic carriage, intermittent shedding, diagnostic limitations, and fragmented surveillance. Its rough LPS phenotype reduces detection by standard smooth-antigen brucellosis assays, while available *B. canis*-specific tests vary in performance and require careful interpretation. Human infection appears uncommon but is likely underdiagnosed, particularly when clinicians do not consider dog exposure or when laboratories rely on tests designed for smooth *Brucella* species.

Effective control requires a One Health approach linking veterinary medicine, human healthcare, diagnostic laboratories, public health agencies, breeders, shelters, and dog owners. The most urgent needs are standardized diagnostic algorithms, improved antigen-specific assays, stronger surveillance, regulation of high-risk dog movement, and realistic management strategies for infected dogs. Addressing these gaps will improve canine welfare, reduce zoonotic risk, and preserve the human–animal bond through evidence-based prevention rather than reactive crisis management.

## AI declaration

The authors declare that they did not use artificial intelligence tools to write this article.

They declare that they used EditAI and Grammar for stylistic and grammatical corrections.

## CRediT authorship contribution statement

**Chiara Storoni:** Writing – original draft, Conceptualization. **Renato Colognato:** Writing – review & editing, Writing – original draft, Conceptualization. **Anna-Rita Attili:** Writing – original draft, Visualization, Resources. **Silvia Preziuso:** Writing – original draft, Visualization, Resources. **Yubao Li:** Writing – review & editing, Supervision. **Vincenzo Cuteri:** Writing – review & editing, Conceptualization.

## Funding sources

This research did not receive any specific grant from funding agencies in the public, commercial, or not-for-profit sectors.

## Declaration of competing interest

“The Authors have nothing to declare”.

## Data Availability

No data was used for the research described in the article.
